# Combining motivational and volitional strategies to promote unsupervised walking in patients with fibromyalgia: study protocol for a randomized controlled trial

**DOI:** 10.1186/1745-6215-15-120

**Published:** 2014-04-11

**Authors:** María-Ángeles Pastor, Sofía López-Roig, Ana Lledó, Cecilia Peñacoba, Lilian Velasco, Inge Schweiger-Gallo, Margarita Cigarán, Carmen Écija, Ramón Limón, Yolanda Sanz

**Affiliations:** 1 Department of Health Psychology, Universidad Miguel Hernández, Crta. Nacional 332, s/n, Sant Joan D'Alacant 03550, Spain; 2 Department of Psychology, Universidad Rey Juan Carlos, Campus de Alcorcón, 28922 Alcorcón, Spain; 3 Department of Social Psychology, Universidad Complutense, Campus de Somosaguas, 28223 Pozuelo de Alarcón, Madrid, Spain; 4 Servicio de Medicina Preventiva y Calidad Asistencial. Hospital de la Plana, Ctra. de Vila-real a Burriana km. 0,5, 12540 Vila-real, Castellón, Spain

**Keywords:** Fibromyalgia, Implementation intention, Intervention, Theory of planned behavior, Walking

## Abstract

**Background:**

Fibromyalgia patients are often advised to engage in regular low- to moderate-intensity physical exercise. The need of fibromyalgia patients to walk has been stressed in previous research. Behavioral self-regulation theories suggest that a combination of motivational aspects (to develop or strengthen a behavioral intention: Theory of Planned Behavior) and volitional aspects (engagement of intention in behavior: implementation intentions) is more effective than a single intervention. In this paper, we describe a protocol for identifying the motivational processes (using the Theory of Planned Behavior) involved in the practice of walking (phase I) and for studying the efficacy of an intervention that combines motivational and volitional contents to enhance the acquisition and continuation of this exercise behavior (phase II). The paper also shows the characteristics of eligible individuals (women who do not walk) and ineligible populations (women who walk or do not walk because of comorbidity without medical recommendation to walk). Both groups consist of members of any of four patients’ associations in Spain who are between 18 and 70 years of age and meet the London Fibromyalgia Epidemiology Study Screening Questionnaire criteria for fibromyalgia. Furthermore, using this study protocol, we will explore the characteristics of participants (eligible women who agreed to participate in the study) and nonparticipants (eligible women who refused to participate).

**Methods/design:**

Two studies will be conducted: Phase I will be a cross-sectional study, and phase II will be a triple-blind, randomized longitudinal study with two treatment groups and one active control group. The questionnaires were sent to a total of 2,227 members of four patients’ associations in Spain. A total of 920 participants with fibromyalgia returned the questionnaires, and 582 were ultimately selected to participate.

**Discussion:**

The first data gathered have allowed us to identify the characteristics of the study population and they support the appropriateness of the inclusion criteria.. When the study is complete, the results will enable us to establish whether this kind of intervention can be used as a self-regulation tool for increasing and maintaining walking as unsupervised physical exercise of low to moderate intensity in fibromyalgia patients.

**Trial registration:**

Trial registration number: ISRCTN68584893

## Background

Fibromyalgia (FM) is a complex chronic condition characterized by widespread musculoskeletal pain, fatigue, sleeping problems and other symptoms with no well-established etiology. Physical exercise is a component of effective treatment [1], and aerobic exercise in particular has been shown to lead to improvements in various health outcomes [2-7]. To the best of our knowledge, most studies conducted to date have included supervised physical exercise programs, which are often associated with low patient adherence [8-12]. In fact, one of the main challenges in FM treatment is achieving long-term adherence to maintain the positive effects of physical exercise on patients’ health [10,13]. Some authors have suggested that adherence represents a main issue and should be a mandatory focus in studies of physical exercise [6]. Walking is a low- to moderate-intensity exercise which has been shown to have positive effects on FM patients’ health status [4,14,15] and is a well-established aerobic activity for these patients [10]. It is also a simple recommendation that promotes patients’ self-management.

Any behavior, such as walking, is more likely to be practiced if people are motivated and develop strategies to engage in it. In the general population, combining motivational and volitional interventions to increase physical exercise or walking are more effective than initiating and promoting each of them separately [16-20]. Motivational intervention encourages individuals to form an intention, and volitional intervention encourages them to form a plan stating when, where and how they will carry out their intention (implementation intentions). In FM patients, however, this combination has not been tested. It is important to examine this issue in this population because the presence of symptoms such as pain and fatigue, which are associated with FM, could affect walking behavior. Furthermore, these symptoms may influence both motivational and volitional processes for starting and maintaining walking behavior in FM patients, which is not the case in the general population. Recently, Ang *et al*. [21] applied an intervention using motivational interviewing to increase supervised walking in FM patients. As these authors underlined, this strategy is not based on any specific theory of health behavioral change. In spite of the good results in the short-term, they concluded that motivational interviewing alone is insufficient to increase physical activity in the long term. These results support the importance of using volitional strategies as well as basing the intervention on well-established theories of health behaviors.

The Theory of Planned Behavior (TPB) [22-24] is a well-established theory in health behavior and has been shown to have predictive power regarding walking and physical exercise [25,26]. In FM patients, TPB constructs (see the “Interventions” subsection below) explained 32% of the variance in the intention to exercise [27]. However, a strong behavioral intention is necessary, but not sufficient, to implement action. The implementation intentions are a theoretical proposal that focuses on the gap between intention and action [28]. Implementation intentions are specific plans that enhance the transition of goal intentions into actions. Motivation is the starting point for behavioral change and volitional strategies, based on implementation intentions, to increase the likelihood of this change [18]. Once the behavior is started, the processes associated with implementation intentions (automatic activation of the behavior when environmental cues are present) are expected to promote maintenance of the behavior because it neither consumes cognitive resources nor produces ego depletion as other self-regulation processes do. Moreover, we expect that the benefits on health status associated with walking behavior will also promote its maintenance in the long term. In other populations and behaviors, the implementation intentions approach has produced better long-term behavioral change [29]. Thus, we expect to find similar effects in our FM population.

Applying both theories to promote behavioral change in FM is expected to allow us to cover two basic processes: (1) the adoption or strengthening of behavioral intentions (motivational process) and (2) their effects on behaviors (volitional or postmotivational process). In the FM context, some special features, such as pain, health status impact and fear of movement, should also be considered [30]. These factors can influence both the intention to exercise and the exercise behavior itself. In fact, some factors have been observed to be barriers to walking (MAP, SLR, YS, CP, LV, MC, AL and CE, unpublished manuscript). Not taking these factors into account has been considered a limitation of previous studies [21].

To increase walking as unsupervised physical exercise, it is important to test whether an intervention which includes motivational strategies (intentions) and volitional strategies (implementation intentions) will be effective. This paper describes the two phases of a protocol. In phase I, we will identify the motivational processes involved in intentions of performing a recommended walking pattern in FM patients, using the TPB and considering the above-mentioned specific FM factors. In phase II, to establish the efficacy of an intervention that combines both motivational processes (based on the results of phase I) and volitional processes to enhance the short-, medium- and long-term adherence to a walking program.

This paper also describes the characteristics of eligible populations (women belonging to FM associations, between 18 and 70 years of age, meeting the London Fibromyalgia Epidemiology Study Screening Questionnaire criteria (London-4) (see http://www.aafplearninglink.org/Resources/Upload/File/AAFP-10-106-London%20Screening-09-07-10.pdf) for FM and unable to walk) and noneligible populations (same characteristics mentioned above, but either able to walk or do not walk because of comorbidity without medical recommendation for walking). This aim is needed to test the similarities between both populations in order to ensure the external validity of the study. We will also explore the characteristics of participants (women from the eligible population who agreed to participate in the study) and nonparticipants (women who refused to participate).

## Methods/design

This study has been approved by the Research Ethics Board of the Miguel Hernández University, and we obtained informed consent from each participant. Furthermore, participants signed a commitment of confidentiality regarding the content of the assigned treatment.

### Description of the overall project

The study consists of two phases, both based on the same population.

Phase I, conducted over the course of 1 year, is a cross-sectional study with two measurement stages and the following three aims:

1. We will seek to gain access to the reference population, identify the eligible population, select the study population and identify the prevalence of our selected walking criteria (stage 1). The results of stage 1 are included in this paper.

2. We will seek to identify the predictors of the behavioral intention to carry out the selected walking program using the TPB and exploring the role of pain, impact of FM and fear of movement (stage 2).

3. We will seek to explore the concordance of walking behavior measurements (subjective and objective measures) (stage 2).

Phase II, which will be conducted over the course of 2 years, is an experimental, randomized, triple-blind study with two treatment groups and one active control group. Longitudinal measures will be taken at baseline and at 7 weeks, 3 months and 9 months of postexperimental intervention. One group will receive a motivational plus implementation intentions intervention (MIG), a second group will receive only an implementation intention intervention (IG) and the control group (CG) will be given a neutral task related to postural hygiene. The three groups will receive the same information on the benefits of physical exercise in FM. Motivational intervention will be based on the predictors of behavioral intention as measured and identified in phase I (stage 2) in order to create or strengthen the behavioral intention during the experimental study.

This triple-blind study includes masking of the following groups in the manner described. Participants will know only that there are different intervention sessions but will not know the session contents; moreover, they will be unaware of the assumptions regarding the superiority of one intervention over another. Measures will be taken by researchers who do not participate in the treatment application and who are blinded to the experimental conditions. The statistician will also be blinded to the study protocol. The researchers who apply implementation intentions will also be blinded with respect to the MIG or IG experimental condition. In this phase, we aim to study the efficacy of the MIG intervention on unsupervised walking over the short term (7 weeks), medium term (3 months) and long term (9 months). The measurement at 7 weeks will be taken during the week after completion of the 6-week minimum walking program. We will base the selection of the medium- and long-term measurements on the assumption that the highest percentage of dropouts occurs between 3 and 6 months in supervised physical exercise programs [8,9]. On the day of the intervention, a researcher will be responsible for the distribution of the participants into the three different treatment groups following random assignment. The researcher will remind participants of their confidentiality commitment.

Our main hypothesis is that a combined intervention (both motivational and volitional) will significantly increase walking behavior in comparison to the control group and that this effect will be higher than that in a merely volitional intervention in the short term and stable in the medium and long term.

Our selected walking pattern for FM patients is between two and four times weekly for about 50 minutes in bouts of 15 to 20 minutes, with a small rest between bouts to allow patients to avoid fatigue and continue the activity, for a minimum of six consecutive weeks [31]. We chose this pattern because it includes several components which will encourage FM patients to do unsupervised physical exercise. Walking is an accessible, cheap and flexible activity; it includes resting; and it requires minimal time, which aids habit acquisition. Although we selected this pattern, we reduced the minimum daily time to 30 minutes (in two bouts of 15 minutes each) and at least two times weekly because our target population is sedentary. In addition, patients will be advised to start physical exercise gradually [10,11], beginning with a low, comfortable intensity according to differences in physical capacity [4]. It is well known that FM patients can benefit from low-intensity walking programs 2 or 3 days per week [32]. Moreover, 30 minutes of continued physical activity has also been shown to have positive effects on health in the general population [33].

### Study population and recruitment process

The fibromyalgia associations of Alicante (ADEFA), Elche (AFEFE), Madrid (AFIBROM) and Talavera de la Reina (AFIBROTAR) collectively comprise a total of 2,438 members with a clinical diagnosis of FM (75.8% diagnosed by rheumatologists, 9.2% by general practitioners, 5.3% by traumatologists, 2.2% by clinical rehabilitation specialists, 0.7% by neurologists and 6.7% by clinicians in other specialties). Slightly more than half (51.4%) of the sample are receiving psychological treatment because of FM. To select our reference population (women between 18 and 70 years of age who meet the London Fibromyalgia Epidemiology Study Screening Questionnaire criteria for fibromyalgia (London-4) ), we first used the associations’ records to select the members who met the two first criteria (*n* = 2,227). We then sent letters to these 2,227 women with information about the study, together with informed consent forms, the London Fibromyalgia Epidemiology Study Screening Questionnaire [34,35] and other questionnaires covering the remaining variables related to the sample characteristics and participation criteria. As we do not have a second clinical diagnosis confirmation, the London-4 criteria were used to ensure population homogeneity. Although these criteria screen only for widespread musculoskeletal pain and do not take into consideration other clinical aspects of FM, they give an optimal sensitivity (100%) in FM screening population studies and good positive predictive values for women in rheumatology settings [35].

**Figure 1:**
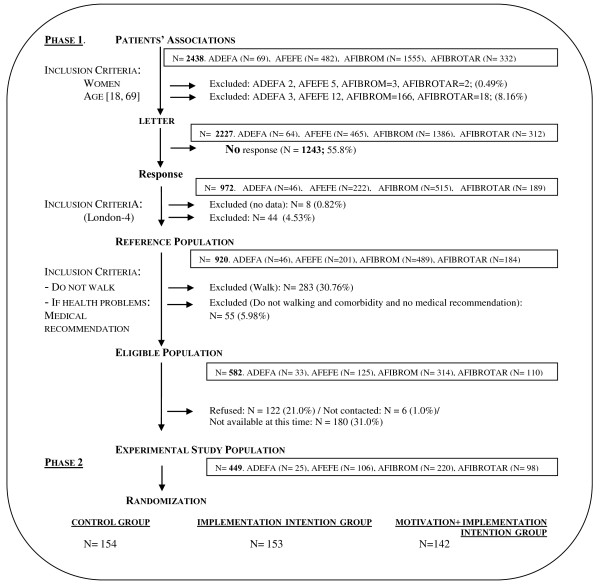
**Flowchart of the participant recruitment process.** ADEFA: Fibromyalgia Association of Alicante; AFEFE: Fibromyalgia Association of Elche; AFIBROM: Fibromyalgia Association of Madrid; AFIBROTAR: Fibromyalgia Association of Talavera de la Reina (Toledo); London-4: London Fibromyalgia Epidemiology Study Screening Questionnaire criteria for fibromyalgia.

A total of 972 questionnaires (43.5%) were returned in two runs. The eligibility criteria were then applied, and our selected reference population subsequently consisted of 920 members with FM (44 did not fit the London-4 criteria and 8 questionnaires did not contain enough data). The remaining criteria were that the individual did not walk or the walking pattern did not comply with one or more features of the selected walking program, the individual was without comorbidity which impeded walking or, if there was comorbidity, the individual had received medical advice to walk. A total of 582 members (63.3%) satisfied these criteria. Figure 1 is a flowchart of the participant recruitment process.

#### Sample size calculation

We calculated the sample size for the experimental study by taking into consideration the 582 FM participants previously identified as the eligible population derived from the different associations: ADEFA (*n* = 33, 5.7%), AFEFE (*n* = 125, 21.5%), AFIBROM (*n* = 314, 54.0%) and AFIBROTAR (*n* = 110, 18.9%). The expected differential increase in the selected exercise prevalence from baseline is 10% for CG, 20% for IG and 40% for MIG. Given the rule that the frequency of dropouts should not exceed the frequency of the effect, we expect a maximum dropout rate of 30% before random allocation. This percentage will be added to the calculated sample size. We have defined the risk α as 5% and the study power as 80%. The minimum magnitude of the expected effect (in relation to the effect on CG) is 10% for IG and 30% for MIG. After we apply these conditions and dividing the sample population into three experimental groups, each group will consist of 115 persons, which is equal to 345 participants. In previously reported supervised programs of moderate-intensity walking, adherence has ranged from 62% [14] to only 37.5% [32]. In other studies, the percentage of patients who finished the supervised program was greater than 70% to 80% [7,15,36-39]. In other words, studies have shown that more than 20% to 60% of patients do not complete physical activity programs which include walking. Therefore, we added a 30% dropout rate as a reasonable calculation, which gives us a sample size of 449 women with FM.

#### Randomization

We used a computer program to randomize the three experimental groups and four fibromyalgia associations, taking into account the size of each center [40]. The 449 participants were allocated to the three experimental groups as follows: CG = 154, IG = 153 and MIG = 142 (Table 1). We applied another computer program to generate random sequences to select the participants from each fibromyalgia association for the experimental and control groups [41]. The randomization results will be checked by analyzing potential confounders such as walking patterns, physical activity, impact of FM, pain, emotional status and age (see the “Statistical analysis” subsection below).

**Table 1:** **Randomized experimental group-center assignments**^**a**^

**Patient associations**	**Assignment, *n *** **(%)**			**Total**
	**CG**	**IG**	**MIG**	
ADEFA	7 (28.0)	9 (36.0)	9 (36.0)	25 (100)
AFEFE	42 (39.6)	29 (27.4)	35 (33.0)	106 (100)
AFIBROM	73 (33.2)	87 (39.5)	60 (27.3)	220 (100)
AFIBROTAR	32 (32.7)	28 (28.6)	38 (38.8)	98 (100)
Total	154 (34.3)	153 (34.1)	142 (31.8)	449 (100)

^a^ADEFA: Fibromyalgia Association of Alicante; AFEFE: Fibromyalgia Association of Elche; AFIBROM: Fibromyalgia Association of Madrid; AFIBROTAR: Fibromyalgia Association of Talavera de la Reina (Toledo); CG: Control group; IG: Implementation intention group; MIG: Motivation and implementation intention group.

### Interventions

**Figure 2:**
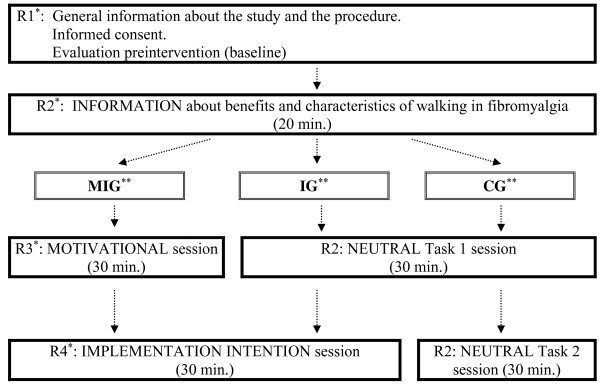
**Schedule for the intervention day.** *R1 is the researcher who gives general instructions and distributes participants in each session. R2 is the researcher who gives information about walking to all participants and applies the control intervention. R3 is the researcher who applies the motivational intervention. R4 is the researcher who applies the implementation intention intervention and is blinded to participants’ previous experiment condition (motivational or neutral). **MIG: Motivation and implementation intention group; IG: Implementation intention group; CG: Control group.

In previous studies in the reviewed literature, we found that interventions for the promotion of walking have been heterogeneous and that no single method has proved to be more effective than others [33]. One of the main advantages of our study is that the intervention is based on two well-established theories for predicting behavior. Following the procedures described in previous research [42,43], all participants will receive information about the benefits of physical exercise in relation to their FM, and they will be asked to engage in the selected walking criteria. In addition, MIG participants will receive motivational interventions, and both the MIG and IG groups will receive information on the positive effects of making plans, specifying (1) days, (2) specific times of day, (3) location (for example, close to work, around the neighborhood) and (4) duration of the exercise (starting from the established minimum). Thus, they will be requested to write a specific individualized plan. The estimated duration of the intervention in each group is 90 minutes, conducted in only one group session with a maximum of 10 people. Figure 2 shows the schedule for the intervention day.

#### Motivational intervention

**Figure 3:**
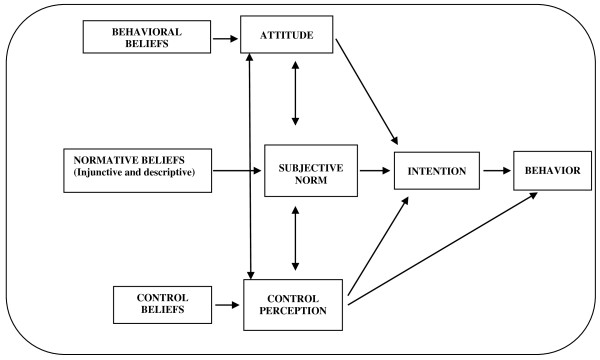
Schematic illustrating the Theory of Planned Behavior.

The aim of the motivational intervention is to ensure strong behavioral intention. The TPB [24] establishes that the behavioral intention (that is, the readiness to perform a specific behavior) is determined by the person’s attitude toward the behavior (the positive or negative global evaluation of performing the specific behavior), the subjective norm (the social pressure perception of engaging in the behavior or not) and the perceived behavioral control (the perception that the behavior is under the person’s control). Attitudes, subjective norms and perceived behavioral control are explained, respectively, by behavioral beliefs, normative beliefs and control beliefs (Figure 3).

In the TPB, beliefs represent the substantive information available to guide people’s decisions with regard to their behavioral performance. Thus, we will target a modification of participants’ beliefs in order to achieve a change with regard to the intention. The motivational intervention will be designed to produce changes in the behavioral, normative or control beliefs associated with the predictors of the behavioral intention (stage 2 of phase I of the research). With regard to the physical exercise behavior, attitude and control perception have been found to be the main predictors of the intention to engage in exercise [25-27]. However, the TPB establishes that the relative weight of each predictor (and consequently the targeted beliefs) changes if the specific behavior changes. In fact, with regard to walking, the control perception has more weight than attitudes [16]. This situation could differ in people with a chronic health problem such as FM. Therefore, it is necessary to explore the particular predictive relationships between the targeted walking behavior and the specific population [24].

A specific proposal for a brief intervention based on the TPB for interventions on walking was recently published [44]. The authors propose several steps for the intervention, such as the identification of the target constructs, the elicitation of key salient beliefs underpinning these constructs and the selection of appropriate behavior change techniques. We followed these recommendations, and phase I (stage 2) of the study is focused on the identification of the target constructs and the salient beliefs through an exploration of the TPB predictors of walking behavior. The beliefs associated with these predictors will be used to enhance intention by implementing writing and imagery techniques. Strategies such as a graded activity will be implemented [45] as soon as we establish the role of pain, FM impact and fear of movement on the behavioral intention in the whole group.

#### Volitive intervention: implementation intentions

Implementation intentions are “if–then” plans that specify when, where and how a goal will be achieved, linking a critical situation (the “if” component) with a goal-directed behavior (the “then” component) [28]. Implementation intentions are hierarchically subordinated to goal intentions (in this study, the behavioral intention). Implementation intentions have been shown to have a moderate to strong effect on goal attainment [46].

We expect participants in the MIG and IG groups to be able to use their plans to adjust their walking activity to the circumstances they have foreseen and specified. We will focus these plans on the management of the inhibitors of the walking behavior, which we will have previously identified in stage 2 of phase I (control beliefs). As has been shown in previous research [47,48], participants can regulate their emotional responses through attentional implementation intentions (for example, to ignore some stimuli) or by specifying their desired behavioral and emotional responses (for example, to be calm or relaxed). Participants form their plans individually, and, in order to facilitate the internalization and memorization of these plans, they will be requested to write the specific implementation intentions on a sheet of paper and place it in a visible location at home. A copy of the sheet will be given to the research team so that the team can perform the follow-up on the specific goals. This will be the last intervention of the day, and the researcher who conducts this session will ask participants to carry on with the task until they finish.

### Measures

#### Phase I (first year)

In the first year of the study, measurements of physical activity, walking behavior, TPB variables, fear of movement, pain, FM impact and emotional status will be taken. All questionnaires will be self-administered by the participants. A summary of variables and schedules in this phase is shown in Table 2.

**Table 2:** **Measures taken and schedule during phase I**^**a**^

**Variables**	**Instrument**	**Schedule**^**b**^		
			**Stage 2**	
		**Stage 1**	**Time 1**	**Time 2 (week 7)**
Walking behavior				
Walking pattern	*Ad hoc* self-report scale	X	X	X
	Daily logs			X
Minutes spent walking in previous week	IPAQ walking	X	X	
Steps and distance	Pedometer			X
TPB variables	*Ad hoc* TPB questionnaire			
Behavioral intention			X	
Attitude			X	
Perceived norm			X	
Perceived behavioral control			X	
Behavioral beliefs			X	X^c^
Injunctive and descriptive beliefs			X	X^c^
Control beliefs			X	X^c^
Fear avoidance	Spanish adaptation of TSK		X	
Total physical activity	IPAQ–Spanish Short Form	X	X	
Intense physical activity		X	X	
Moderate physical activity		X	X	
Sitting		X	X	
Fibromyalgia impact	Spanish adaptation of FIQ		X	
Pain intensity	11-points numerical rating scale	X	X	
Emotional status: Distress	Spanish adaptation of HADS		X	

^a^FIQ: Fibromyalgia Impact Questionnaire; HADS: Hospital Anxiety and Depression Scale; IPAQ: International Physical Activity Questionnaire; TPB: Theory of Planned Behavior; TSK: Tampa Scale for Kinesiophobia. ^b^Women who attended the association or university laboratory for assessment. Daily logs and pedometer readings were taken at time 1. ^c^For test–retest reliability.

##### Behavioral variables

To assess for behavioral variables, we will gather participants’ self-reported information as outlined below.

1. *Spanish version of the International Physical Activity Questionnaire–Short Form (IPAQ-S)*[49]: The IPAQ-S is an instrument designed to assess physical activity among adults. It can be used as a tool in intervention studies, although it was not designed for this purpose. There is a lack of self-report measures of physical activity designed for FM patients. In addition, the results obtained from well-established instruments in general populations are not good enough in FM patients [50]. However, the IPAQ-S was previously found to be better than other physical activity instruments in identifying sedentary patients with chronic fatigue syndrome [51]. We have chosen to use the IPAQ-S because of its Spanish translation; its short form, which is an advantage for our first mailed survey and for the FM population; and because it includes walking as a type of physical activity. The IPAQ-S allows respondents to record their sitting, walking, moderate-intensity and vigorous activities in minutes per week and calculate a total physical activity score. We will use the median minutes per week for each activity for assessment purposes. The rules for cleaning and correcting data will be applied [49]. In this phase, we will use the IPAQ-S to characterize our reference and study populations.

2. *Behaviors*: We will use four measures to assess walking behavior. First, we will use a self-report scale to assess the usual walking pattern to identify the study population. The scale consists of six questions designed to assess whether participants usually walk for physical exercise, how many minutes they walk daily, how many days per week they walk, how many consecutive weeks they walked, whether they took rests during walking and whether they had been advised to walk by their doctor.

Second, we selected the walking score from the IPAQ-S [49] because it refers to the previous week of the assessment. It can thus provide a more accurate measurement than our general measures because of its proximity in time and because it can limit the influence of the participant’s memory.

Third, we have designed weekly and daily logs to assess walking behavior over the course of 6 consecutive weeks. Daily logs include the days and minutes of walking and the duration of rest during the activity. In addition, participants score the perceived intensity of the activity using the Borg Rating of Perceived Exertion scale [52]. Participants will use a pedometer (3D USB Pedometer PDM-2608) to record the number of steps and distance covered over the same period.

These measures will be used to explore the agreement between subjective and objective measurements. We need to establish this agreement because the entire study population will use daily logs, but only half of them will use pedometers.

##### Motivational variables

To assess for motivational variables, we will gather participants’ self-reported information as outlined below.

1. *TPB variables*: Behavioral intentions, attitudes toward behaviors, perceived norms, perceived behavioral control, behavioral beliefs, normative beliefs and control beliefs will be measured according to the TPB recommendations [24]. The questionnaire was tested in a previous study and showed good psychometric properties (MAP, SLR, YS, CP, LV, MC, AL and CE, unpublished manuscript). These measures will allow us to identify the predictors of behavioral intentions and consequently to modify or strengthen the beliefs upon which they are based during the motivational interventions.

2. *Fear of movement*: We will use the total score on the Spanish adaptation of the Tampa Scale for Kinesiophobia to measure fear of movement [53]. Patients will rate 11 items on a four-point Likert scale (from 1 = totally disagree to 4 = totally agree). This measure will allow us to explore the role of fear of movement in relation to behavioral intentions.

##### Health status variables

To assess for health status variables, we will gather participants’ self-reported information as outlined below.

1. *Pain*: We will use the total score on an 11-point numerical rating scale (from 0 = “no pain at all” to 10 = “the worst pain you can imagine”) adapted from Jensen *et al*. [54] to assess the maximum, minimum and usual pain intensity during the week prior to the evaluation and the pain intensity that moment. The scale has shown good psychometric properties in Spanish FM samples [55,56].

2. *FM impact*: We will use the total score of the consensus version of the Spanish adaptation of the Fibromyalgia Impact Questionnaire (FIQ), which is valid and reliable [57].

3. *Emotional status*: The perceived total distress is measured with the Spanish adaptation [58] of the Hospital Anxiety and Depression Scale [59]. Patients rate 14 items on a four-point answer scale. This scale has been shown to be valid and reliable [58].

Our aim in assessing these variables is, on the one hand, to explore their role in behavioral intention and, on the other hand, to explore them as potential confounders.

#### Phase II (second and third years)

A summary of variables and the schedule of phase II is given in Table 3.

**Table 3:** **Measures and schedule of phase II**^**a**^

	**Instrument**	**Schedule**			
**Outcomes**		**Baseline**	**Week 7**	**Week 20 (3 months)**	**Week 43 (9 months)**
*Primary*					
Walking pattern	*Ad hoc* self-report scale	X	X	X	X
	Daily logs		X	X	X
Steps and distance	Pedometer^b^		X	X	X
*Secondary*					
Total physical activity	IPAQ	X	X	X	X
Intense physical activity		X	X	X	X
Moderate physical activity		X	X	X	X
Walking		X	X	X	X
Sitting		X	X	X	X
Physical function	Six-Minute Walk Test	X	X	X	X
FM Impact	FIQ	X	X	X	X
Pain intensity	*Ad hoc* scale	X	X	X	X
Emotional status: Distress	HADS	X	X	X	X
*Mediators*					
Behavioral intention^c^	TPB items	X	X	X	X
Implementation intentions^c^	*Ad hoc* items		X	X	X

^a^FIQ: Fibromyalgia Impact Questionnaire; HADS: Hospital Anxiety and Depression Scale; IPAQ: International Physical Activity Questionnaire; TPB: Theory of Planned Behavior. ^b^Limit of recording is 60 days. ^c^Also measured at the end of the intervention sessions.

#### Primary outcome measures

Two primary outcomes will be assessed as indicators of each intervention’s effect on exercise adherence:

1. We will measure the proportion of participants who perform the full minimum walking criteria (at least 30 minutes in bouts of 15 minutes with a small rest between bouts, twice times weekly over a minimum of 6 consecutive weeks) at the end of the 6-week period and the proportion of participants who have maintained it at 3 and 9 months. The expected efficacy of MIG is 30% higher than CG and 10% higher than IG.

2. Among participants who perform the full minimum walking criteria, we will focus on the proportion who reach the recommended pattern for FM patients at week 6 (between two and four times weekly for about 50 minutes in bouts of 15 to 20 minutes over a minimum of 6 consecutive weeks) [31].

We will use the same self-reported measurements of the behavior from phase I (*ad hoc* self-reported items and daily logs) and pedometer readings.

#### Secondary outcome measures

*Six-Minute Walk Test (6MWT)*: The 6MWT is a clinically relevant measure of the physical function that the Spanish Society of Rheumatology recommends using with FM patients [60].

The remaining secondary measures are described in the measures of phase I: IPAQ-S score [49], FIQ score [57], pain intensity rating [55] and emotional status [58].

#### Mediators

1. *Behavioral intentions*: In a previous study, we designed and proved the high internal consistency (α = 0.87) of five items for an *ad hoc* questionnaire (MAP, SLR, YS, CP, LV, MC, AL and CE, unpublished manuscript). These items are used to assess the individual’s readiness to carry out the full walking pattern: “I intend to walk,” “I will walk,” “I am willing to walk,” “I plan to walk” and “I will make an effort to walk at least 30 minutes in bouts of 15 minutes, with a small rest between bouts, twice weekly for a minimum of 6 consecutive weeks.” All answers given are scored according to a numerical rating scale from 1 to 7.

2. *Implementation intentions*: A postexperiment questionnaire will be used to assess how committed patients felt toward meeting their goals (“How committed did you feel to the self-regulation intention?” and “How much did you try to achieve your self-regulation intentions?”) and their perceived performance (“How difficult was it to achieve your walking behavior?” “Did your self-regulation intention help you achieve the proposed walking behavior?” and “How well did you succeed in realizing your self-regulation intention?”). All of these items will be accompanied by 11-point answer scales ranging from 0 (“not at all”) to 10 (“very”).

#### Statistical analysis

Data will be entered into a relational database system (Microsoft Access; Microsoft, Redmond, WA, USA) with range rules and forms, which will reduce the number of data entry mistakes. In addition, we will apply a program to check and clean data [61]. Analyses will be made using the IBM SPSS Statistics 21 software package (SPSS, Chicago, IL, USA) and LISREL software (SSI Scientific Software International, Skokie, IL, USA).

#### Phase I

Descriptive analyses have been performed. The sociodemographic characteristics, symptoms, walking and physical activity of eligible and noneligible populations have been compared by conducting χ^2^ tests, *t*-tests, analysis of variance and median nonparametric tests, depending on the characteristics of the variables (Table 4). These comparisons were conducted to ensure that both samples differ only with regard to the inclusion criteria of the study. The same analyses were performed to compare participants with individuals who refused to participate and nonresponders (that is, not contacted or not available at the time of measurement) (Table 5).

**Table 4:** **Descriptive statistics in eligible and noneligible populations**^**a**^

**Variables**	**Noneligible**					**Eligible**				
	**Mean**	**(95% CI)**	**SD**	**Median**	**(95% CI)**	**Mean**	**(95% CI)**	**SD**	**Median**	**(95% CI)**
Age (years)	53.51	(52.59 to 54.42)	8.52	54.24		52.14	(51.41 to 52.87)	8.96	52.91	
Pain intensity	6.63	(6.43 to 6.83)	1.82	6.62		6.73	(6.59 to 6.87)	1.70	6.75	
Fatigue	8.01	(7.79 to 8.23)	2.01	8.00		8.20	(8.36 to 8.39)	1.96	9.00	
Fatigue upon waking	7.99	(7.74 to 8.25)	2.37	9.00		8.17	(8.00 to 8.25)	2.20	9.00	
Fatigue impact perception	6.78	(6.48 to 7.08)	2.77	7.00		7.01	(6.78 to 7.23)	2.72	7.00	
Cognitive problems	6.77	(6.48 to 7.05)	2.62	7.00		7.00	(6.78 to 7.21)	2.66	8.00	
Sleeping disorder impact perception	7.07	(6.75 to 7.38)	2.94	8.00		7.02	(6.70 to 7.26)	2.95	8.00	
Cognitive problems impact perception	6.30	(6.00 to 6.61)	2.83	7.00		6.57	(6.34 to 6.80)	2.83	7.00	
IPAQ-Sitting, mi/wk				240.5	(240.5 to 300.5)				300.5	(240.5 to 300.5)
IPAQ-Walking, min/wk				300.5	(245.5 to 350.5)				180.5	(140.5 to 180.5)
IPAQ-Moderate activities, min/wk				0.5	(0.5 to 60.5)				0.5	(0.5 to 0.5)
IPAQ-Vigorous activities, min/wk				0.5	(0.5 to 0.5)				0.5	(0.5 to 0.5)
IPAQ-Total activities, min/wk				383.5	(300.5 to 420.5)				180.5	(150.5 to 210.5)
	***n***	**(%)**				***n***	**(%)**			
Education level										
Literate	42	(12.5)				67	(11.5)			
Primary	138	(40.9)				261	(44.8)			
Secondary	106	(31.5)				163	(28.0)			
University	51	(15.1)				91	(15.6)			
Employment status										
Working	88	(26.3)				177	(30.5)			
Unemployed	63	(18.8)				118	(20.4)			
Retired	26	(7.8)				38	(6.6)			
Retired (pain)	39	(11.6)				59	(10.2)			
Sick leave	32	(9.6)				50	(8.6)			
Housewife	87	(26.0)				138	(23.8)			

^a^CI: Confidence interval; IPAQ: International Physical Activity Questionnaire; SD: Standard deviation.

**Table 5:** **Descriptive statistics in participants and nonparticipants from eligible population**^**a**^

**Variables**	**Nonparticipant group**					**Participant group**				
	**Mean**	**(95% CI)**	**SD**	**Median**	**(95% CI)**	**Mean**	**(95% CI)**	**SD**	**Median**	**(95% CI)**
Age (years)	52.41	(51.42 to 53.40)	8.79	53.17		51.84	(50.75 to 52.93)	9.16	52.69	
Pain intensity	6.76	(6.56 to 6.95)	1.72	6.75		6.70	(6.50 to 6.90)	1.67	6.50	
Fatigue	8.19	(7.96 to 8.42)	2.02	9.00		8.22	(7.99 to 8.44)	1.90	9.00	
Fatigue upon waking	8.24	(7.99 to 8.49)	2.22	9.00		8.10	(7.84 to 8.36)	2.17	9.00	
Fatigue impact perception	7.05	(6.76 to 7.34)	2.60	8.00		6.96	(6.62 to 7.29)	2.85	7.00	
Cognitive problems	6.92	(6.62 to 7.22)	2.69	7.00		7.08	(6.77 to 7.39)	2.63	8.00	
Sleep disorder impact perception	7.01	(6.66 to 7.35)	3.07	8.00		7.03	(6.70 to 7.37)	2.82	8.00	
Cognitive problems impact perception	6.46	(6.14 to 6.79)	2.86	7.00		6.68	(6.35 to 7.02)	2.79	7.00	
IPAQ-Sitting, min/wk				270.5	(240.5 to 300.5)				300.5	(240.5 to 330.5)
IPAQ-Walking, min/wk				150.5	(120.5 to 200.5)				180.5	(120.5 to 210.5)
IPAQ-Moderate activities, min/wk				0.5	(0.5 to 0.5)				0.5	(0.5 to 15.5)
IPAQ-Vigorous activities, min/wk				0.5	(0.5 to 0.5)				0.5	(0.5 to 0.5)
IPAQ-Total activities, min/wk				160.5	(120.5 to 210.5)				210.5	(180.5 to 240.5)
	***n***	**(%)**				**n**	**(%)**			
Education level										
Literate	32	(10.4)				35	(12.8)			
Primary	132	(42.9)				129	(47.1)			
Secondary	86	(27.9)				77	(28.1)			
University	58	(18.8)				33	(12)			
Employment status										
Working	92	(30.4)				85	(31.1)			
Unemployed	59	(18.9)				59	(21.6)			
Retired	25	(8.1)				13	(4.8)			
Retired (pain)	32	(10.4)				27	(9.9)			
Sick leave	32	(10.4)				18	(6.6)			
Housewife	67	(21.8)				71	(26.0)			

^a^CI: Confidence interval; IPAQ: International Physical Activity Questionnaire; SD: Standard deviation.

No significant differences were found between the noneligible population (*n* = 338) and the eligible population (*n* = 582) regarding the distribution of the patients drawn from the different FM associations (χ^2^ = 2,670, *P* = 0.445, df = 3. With regard to sociodemographic variables, we found significant differences only for age between the eligible and the noneligible populations (*t* = 2.264, *P* = 0.024, df = 914, mean difference = 1.37, 95% CI = 0.18 to 2.55). This difference was not found, however, when we considered the FM associations in the analysis (eligible group by FM association: F = 1.928, *P* = 0.124; noneligible group by FM association: F = 2.403, *P* = 0.067). No significant differences were found between groups for either education level (χ^2^ = 1.810, *P* = 0.613, df = 3) or employment status (χ^2^ = 5.912, *P* = 0.436, df = 6). Groups were also similar in pain intensity perception (*t* = -0.833, *P* = 0.415, df = 913), fatigue (*t* = -1.38, *P* = 0.167, df = 909), fatigue upon waking (*t* = -1.16, *P* = 0.245, df = 910), impact perception of fatigue (*t* = -1.21, *P* = 0.226, df = 909), cognitive problems (*t* = -1.27, *P* = 0.205, df = 910), impact perception of cognitive problems (*t* = -1.37, *P* = 0.173, df = 908) and impact perception of sleeping disorders (*t* = 0.25, *P* = 0.806, df = 911). The last six variables were included in the mailed questionnaire to identify the study population. All of them were calculated for the previous week and were measured using an 11-point numerical rating scale (from 0 = nothing at all to 10 = totally). In relation to IPAQ-S variables, median total activity, walking and moderate-intensity activities were different (*P* ≤ 0.01) and similar in vigorous activities (*P* = 0.296) and sitting (*P* = 0.442) (Table 4).

All eligible individuals were contacted (*N* = 582) because of the difficulties involved in the participation of the first 449 selected women, such as the failure to contact them after repeated attempts, nonattendance after three citations and distance of residence from the study location. Ultimately, 274 patients accepted and attended the first evaluation session (participation rate = 47.1%). No significant differences were found between these 274 participants and women with FM who did not participate in this phase of the study (*n* = 308) on the basis of any of the above-mentioned variables (age: *t* = 0.767, *P* = 0.443, df = 579; education level: χ^2^ = 5.567, *P* = 0.135; df = 3; employment status: χ^2^ = 7.567, *P* = 0.272, df = 6; pain intensity perception: *t* = 0.389, *P* = 0.697, df = 579; fatigue: *t* = -0.178; *P* = 0.858, df = 576; fatigue upon waking: *t* = 0.785, *P* = 0.433, df = 576; impact perception of fatigue: *t* = 0.410, *P* = 0.410, df = 577; cognitive problems: *t* = -0.718, *P* = 0.473, df = 577; impact perception of cognitive problems: *t* = -0.939, *P* = 0.348, df = 577; impact perception of sleeping disorders: *t* = -0.107, *P* = 0.915, df = 577). The groups showed different medians of moderate activities (*P* ≤ 0.01), but similar medians in total activity (*P* = 0.066), sitting (*P* = 0.208), walking (*P* = 0.609) and vigorous activities (*P* = 0.115). In addition, the groups presented a proportion of noncompliance similar to that of the total unsupervised pattern (45.8% and 44.2%, respectively) and the same proportion reported for medical recommendation to walk (81.8% in each group) (Table 5).

Internal consistency analysis will be performed for the different scales using the Cronbach’s α coefficient. Path analysis will be conducted to identify the relative weights of attitudes, perceived norms and perceived behavioral control in the prediction of the behavioral intention (path coefficients). Furthermore, we will test the role of the fear of movement, pain and FM impact. Spearman and Pearson correlations and cross-tabulation analysis (depending on the variables) will be used to explore the agreement of the behavioral measurements.

#### Phase II (second and third years)

As our main outcome is a binary variable, backward stepwise logistic regression analysis will be applied. This method will allow us to investigate whether the experimental condition is associated with the acquisition of the walking pattern. Analysis will be performed for each time measure. Changes in walking behavior over time will be analyzed by applying a Cox proportional hazards regression model for recurrent events. Using this methodology, we will estimate the hazard function of the outcome measure (the likelihood of walking over time). We will analyze differences between groups by introducing the experimental condition as a predictor of the moment in the longitudinal study when women maintained the walking pattern.

Randomization results will be checked to ensure that groups are comparable at the beginning of the study in terms of their distribution of potential confounding factors (walking pattern, IPAQ, FIQ, pain, age, emotional status and behavioral intention). The potential confounders will be selected according to previous analyses at baseline by applying confusion criteria (associations with exposition: *P* ≤ 0.20 and correlation coefficients >0.10; associations with the main result in CG: OR < 0.67 or OR > 1.5). Statistical adjustment to control for the impact of confounders on effect estimation will be applied by introducing them into the analyses (if changes in OR are greater than 10%). Effect modifiers of basal walking pattern will also be tested.

Multiple regression analysis will be performed for each secondary outcome variable. Baseline data will be introduced in the predictive model for each time measure. We will test the mediating effects of the behavioral intention and of implementation intentions in the total sample using path analysis.

## Discussion

The benefits of physical activity associated with FM patients’ health status are well established. Exercise programs have been applied in isolation or together with other interventions in multidisciplinary treatments. There is evidence of clinical benefits of aerobic exercise for FM patients [3]. Most exercise programs are supervised in professional contexts, but the reported adherence to these programs is not encouraging [8-12]. Walking represents a similar problem. Although some authors have reported satisfactory compliance rates for walking [14], the exercise was performed in specific conditions, with walking poles and in group sessions with a monitor, which might contribute to an increase in adherence.

Walking is recommended to people with FM [4] and to adults in general [62]. It is easily accessible, cheap and popular, and it helps promote self-regulation and self-efficacy [10]. Our study will endorse these features because, although unsupervised, participants will be expected to walk for a minimum time under specific conditions. This minimum is easily reached by sedentary FM participants, and each participant can increase it gradually (at least up to the recommended pattern for FM patients [31]) or maintain the minimum level established in the study. In fact, because our intervention promotes participants’ self-regulation, we also expect that they will set their own goals, which may include longer walking times. In addition, the study will allow us to test whether different basal walking patterns have differential benefits by analyzing the effect modifier of the relationship between experimental conditions and main results. For example, the effect of interventions might be higher in participants who do not walk at all than in participants who do.

To the best of our knowledge, this study is the first to address adherence to unsupervised walking patterns of FM patients by combining motivational and volitional strategies with strong theoretical foundations in health psychology. This combination has shown good results in the general population [16,18,19], but to our knowledge it has not been used in FM patients.

TPB is the selected theoretical model for the design of the motivational intervention. It has allowed us to identify the modal salient beliefs (behavioral, normative and control) in a previous elicitation study with a sample of the study population (MAP, SLR, YS, CP, LV, MC, AL and CE, unpublished manuscript). As soon as the predictors of the behavioral intention are assessed in phase I, we will address the specific beliefs of FM participants, which should be modified or strengthened in the motivational intervention. By adding to these intervention implementation intentions, we expect to enhance the selected walking behaviors and to raise adherence. Both strategies have been recommended elsewhere [5]. Furthermore, using implementation intentions encourages each participant to carry out their own action plans (based on the established minimum), taking into account their personal circumstances.

The initial data have allowed us to identify the characteristics of the study population. In addition, the preliminary results have shown differences between members of the FM population who meet the inclusion criteria in this study (eligible population) and those who do not (noneligible) in total, moderate and walking activities. These results support the appropriateness of these criteria with regard to walking patterns. The activities and demographic characteristics of the participants who refused to participate, as well as the nonresponders from the eligible population, were similar to those of the participants. Although participants were different in regard to moderate intensity activities, the median values were the same and only a slightly higher percentage of FM participants took part in moderate activity as compared to nonparticipants. These results also support the representativeness of the study sample. We are aware of the difficulties of the research because the targeted population is sedentary. However, the effect of exercise interventions has been substantial in this type of patient.

The study has some limitations, which are mainly related to the voluntary participation of the FM participants from the several FM associations. First, we should mention the selection bias. It is possible that these participants were more active than other FM patients who do not belong to an association. This fact could influence both their physical activity and their motivation to exercise, and it could limit the effect of the intervention on walking behavior. However, this problem can be corrected by the possible variability in the behavioral intentions and the selected target population, who are all sedentary people. Furthermore, in Spain, the majority of FM patients belong to an association because their treatment needs are not covered by the public health system. Joining a FM patient association substantially decreases the expenses related to the illness [63]. Therefore, the variability of the members might be considered similar to the variability of people with FM.

Second, the recruitment of participants from among members of FM associations does not allow us to monitor the diagnostic criteria. The clinical confirmation of the FM diagnosis is not part of our study protocol. However, American College of Rheumatology criteria [64] have been widely used by Spanish physicians, because they are the criteria for FM diagnosis recommended by the Spanish Ministry of Health [65,66] and the Spanish Society of Rheumatology [60]. Moreover, a screening questionnaire [34] was introduced into the study as an inclusion criterion.

Third, we do not know the participants’ level of education regarding exercise in FM. However, they will receive the same information about the benefits of physical exercise and walking, specifically as a component of the intervention.

Fourth, the primary tool we will use to assess physical activity habits has not been validated in FM, but our interest is focused on the performance of a specific walking pattern, which is not measured by standard questionnaires. The IPAQ results will be used mainly to support our population selection based on self-reported walking behavior.

It is important to underline that as soon as the final 274 participants agree to participate, strategies to avoid having participants dropping out will be implemented. In that sense, we will contact the 180 women who failed to attend the evaluation session but did not refuse to participate in the study. Because the participants belong to FM associations which conduct other activities for their members, we may have problems with the masking process. Participants might talk among themselves about the activities performed in each intervention group. However, nobody knows which intervention group is expected to be more successful. Participants will sign a commitment of confidentiality, and, furthermore, we will also assess the shaping of implementation intentions in the control group.

In spite of these limitations, the study addresses an important need: the enhancement of self-management in the treatment of FM. It focuses on increasing adherence to a strategy which has been shown to be effective (physical exercise, specifically walking). Furthermore, the results are expected to show the effectiveness of a short intervention which is easily applicable with a minimum of training and cost and that can be incorporated into routine clinical practice.

## Trial status

Enrollment into the study started in May 2012. The experimental study are expected to be completed by the end of June 2014.

## Abbreviations

CG: Control group; FM: Fibromyalgia; IG: Implementation intention group; MIG: Motivational and implementation intention group; TPB: Theory of Planned Behavior.

## Competing interests

The authors declare that they have no competing interests.

## Authors’ contributions

MAP and SLR were involved with the conception and design of the work, analysis and interpretation of data and manuscript writing. ALL, LV, MC, CE and YS performed data collection and critical revision of the manuscript. CP was involved with the design of the work and critical revision of the manuscript. ISG was involved with the design of the work and manuscript writing. RL analyzed and interpreted the data. All authors read and approved the final version of the manuscript.

## Authors’ information

María-Ángeles Pastor is the Principal Investigator of the study.
